# Amyotrophic Lateral Sclerosis Patients Show Higher Urinary Levels of Lead and Copper: A Pilot Case-Control Study

**DOI:** 10.3390/biomedicines13102385

**Published:** 2025-09-29

**Authors:** Ana Santurtún, Lucía Pérez-Soberón, María José Sedano, Javier Riancho

**Affiliations:** 1 Unit of Legal Medicine, Department of Physiology and Pharmacology, University of Cantabria, IDIVAL, 39011 Santander, Spain; 2 Department of Medicine and Psychiatry, University of Cantabria, 39011 Santander, Spain; 3 Service of Neurology, Hospital Universitario Marques de Valdecilla-IDIVAL, 39008 Santander, Spain; 4 Service of Neurology, Hospital General Sierrallana-IDIVAL, 39300 Torrelavega, Spain; 5 CIBERNED, 28031 Madrid, Spain

**Keywords:** Amyotrophic lateral sclerosis, copper, heavy metals, lead, manganese, selenium, urine, zinc

## Abstract

**Background/Objectives:** Amyotrophic Lateral Sclerosis (ALS) is the most frequent neurodegenerative disease affecting motor neurons. Sporadic ALS cases, which represent over 90% of the total, result from the interaction between genetic predisposition, aging, and environmental factors. Regarding natural environmental risk factors, the analysis of the role of exposure to heavy metals is of particular interest due to the well-known neurological effects of certain compounds. This study aims to compare the levels of heavy metals in urine samples in a cohort of patients with ALS who have not changed their living environment with the levels found in healthy controls (HCs). **Methods:** A cross-sectional case-control (14 patients with ALS vs. 28 HC) observational study was conducted in which urine samples were analyzed for five heavy metals (lead, manganese, selenium, copper, and zinc) using Inductively Coupled Plasma Mass Spectrometry (ICP-MS). **Results:** The patients with ALS showed significantly higher urine levels of lead (*p* < 0.001) and copper (*p* = 0.007) and a subtle increase in manganese concentrations (*p* = 0.043). Urine samples reflect recent exposures, so if the source of metals was related to the residential environment (the patients in the present study had not moved), dietary habits, or certain activities or hobbies that had not changed since diagnosis, it would be representative. **Conclusions:** In this pilot study, patients with ALS presented higher urinary levels of lead, manganese, and copper. Future larger studies are needed to elucidate the precise role of these heavy metals in ALS pathogenesis.

## 1. Introduction

Amyotrophic Lateral Sclerosis (ALS) is the most frequent neurodegenerative disease affecting motor neurons and lacks an effective disease-modifying therapy [1]. It is characterized by progressive muscle denervation that, on average, leads to the patient death 2–3 years after diagnosis, usually related to respiratory complications [2,3,4]. ALS incidence rises from 1 to 3 cases per 100,000 pop/year across Caucasian populations, with lower incidences among Asians and African Americans, and a peak of incidence between 60 and 70 years [1,5,6]. In addition, global prevalence ranges from 4 to 5 cases per 100,000 population [1,4].

ALS can be divided into familial (fALS) and sporadic ALS (sALS). sALS cases, which represent over 90% of the total, result from the interaction between genetic predisposition, aging, and environmental factors. Therefore, when the sum of these elements reaches a determined threshold, ALS onset will occur [4]. Some authors have estimated that sALS heritability is about 60%, whereas the environmental component might represent up to 40% of the process [7]. On this basis, these environmental exposures might be determinant for developing sALS [7]. The rising global incidence of ALS—with projections of a 24% increase by 2040 [8]—and the identification of high-incidence, non-genetic clusters have intensified research into the role of environmental factors in the disease [9]. The term environmental conditions contains a very extensive list of factors, including external and internal ones [10]. Among the latter, several situations have been associated with the disease. In the last years, some groups have been studying the relationship between different lifestyle traditions and ALS development, including smoking, physical activity, and eating habits [4,11]. In addition, some studies have demonstrated the connection between autoimmune diseases and ALS, with a higher risk among patients with type 1 diabetes, multiple sclerosis, myasthenia gravis, and polymyositis [10,12]. Moreover, solid studies suggest a metabolic disturbance in ALS [13,14]. Most of them propose some degree of imbalance between fatty and glucose metabolism in patients with ALS [10]. Contrary to other neurodegenerative diseases, patients with ALS who have hyperglycemia or hyperlipidemia seem to have a better prognosis [1]. Among external environmental conditions potentially promoting ALS development, heavy metals, pollution, and infectious agents have been some of the most studied ones. Regarding natural environmental risk factors, the study of air quality and the analysis of the effects of exposure to toxic substances at the workplace have gained prominence over the past decade [15,16,17,18,19]. In this context, the role of exposure to heavy metals (both through occupational exposure and through outdoor and indoor air) is of particular interest due to the well-known neurological effects of certain compounds [20,21]. To date, the vast majority of studies that have analyzed the role of metal exposure in ALS have focused on occupational exposure [22], on assessments based on lifestyle habits [23], or on the environmental characteristics in which the patients lived [24].

Based on previously reported associations between heavy metal exposure and ALS, we hypothesize that patients with ALS may exhibit elevated levels of heavy metals in biological samples compared to healthy controls (HCs). If confirmed, this finding could reflect not only increased environmental exposure but also potential differences in the metabolism and clearance of heavy metals in individuals with ALS.

Within this context, the present study aims to compare the levels of heavy metals in urine samples from a cohort of patients with ALS who have not changed their living environment with those found in a group of HCs matched by residential area and gender.

## 2. Materials and Methods

A cross-sectional case-control observational study was conducted in Cantabria, a coastal region in northern Spain. This work was approved by the Research Ethics Committee of Cantabria (CEIm of Cantabria; reference number 2023.230). All participants provided their written consent following proper and thorough information.

Participant enrollment followed a 2:1 ratio, with two controls recruited for each case. Regarding case selection, all living patients with ALS who met the predefined inclusion and exclusion criteria were invited to participate. The inclusion criteria for cases were (i) a diagnosis of clinically probable and/or definite ALS according to the Awaji criteria; (ii) age > 18 years; (iii) residence within Health Area I of the Autonomous Community of Cantabria; and (iv) willingness to participate, documented by signed informed consent. The exclusion criteria were (i) presence of renal and/or hepatic disease; (ii) use of nutritional supplements (e.g., magnesium, zinc) or chelation therapy within the previous 6 months; and (iii) inability to provide a urine sample by spontaneous voiding.

For the HCs, the inclusion criteria were (i) age > 55 years; (ii) residence within Health Area I of Cantabria; (iii) absence of neurodegenerative disease; (iv) no genetic or household relationships with patients with ALS; and (v) signed informed consent. The exclusion criteria were the same as those applied to the cases.

The controls were consecutively recruited from the Primary Health Care Centers of Health Area I, corresponding to the same catchment area as the cases. Matching was performed by sex and residential area

To determine the heavy metals urine concentrations of the patients and HCs, all participants were instructed to collect a urine sample in a sterile container. They all had to collect it late in the afternoon of a specific Friday of a given week and keep it frozen (−20 °C). The quantification was carried out at one single time.

Five heavy metals (lead [Pb], manganese [Mn], selenium [Se], copper [Cu], and zinc [Zn]) were analyzed in each sample. For the analysis of the heavy metals in urine samples, Inductively Coupled Plasma Mass Spectrometry (ICP-MS) was used. Heavy metal concentrations were not adjusted for renal clearance, as ALS-related amyotrophy [2] is often linked to low creatinine levels that could bias the results.

Once the urine sample concentrations were obtained, a descriptive analysis was performed, specifying the average, median and 25th and 75th percentiles. Heavy metals concentration units were expressed in μg/L. If no concentration was detected (which occurred in some samples from participants in the detection of manganese and lead), given the sensitivity of the technique (the detection threshold is 0.1 μg/L for lead and 0.5 μg/L for manganese), instead of assigning a value of 0, the midpoint value between the detection threshold and zero was assigned to these samples (0.05 μg/L for lead and 0.25 μg/L for manganese).

Subsequently, in order to assess if differences between groups were significant or not according to the data distribution, either the Mann–Whitney U test or Student’s *t*-test were used. The latter was employed in the case of Se, due to the data showing a parametric distribution. For all the remaining heavy metals, the Mann–Whitney U test was used.

## 3. Results

Forty-two subjects participated in the investigation. In total, 14 patients, 7 men and 7 women (mean age 65 years [range 51 to 84]; 12 spinal onset ALS, 2 bulbar onset ALS) were studied. In addition, two HCs were selected for each case. The HCs were coupled by gender and living area (areas were classified as urban if having a minimum density of 300 inhabitants per km^2^ and a minimum population of 10,000 inhabitants). Thus, a total of 28 HCs (14 men and 14 women), mean age 70 years (range 61 to 92) were assessed. The main clinical-demographic characteristics of the patients with ALS and the HCs are summarized in Table 1.

### 3.1. Lead (Pb)

**Figure 1:**
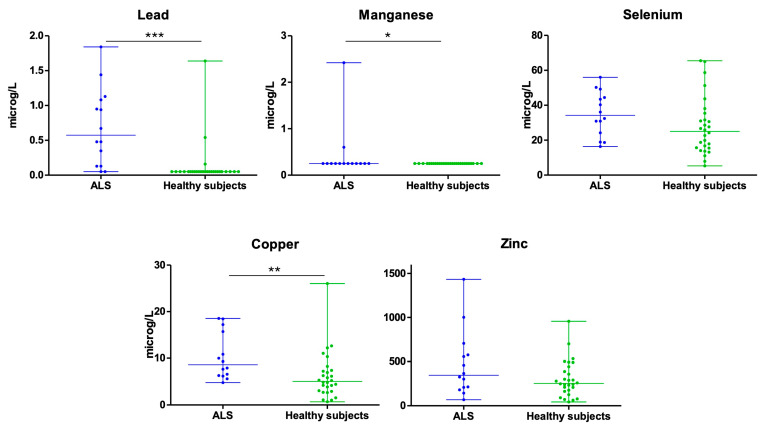
Concentration of heavy metals in urine of patients with ALS and healthy subjects (* *p* < 0.05; ** *p* < 0.005; *** *p* < 0.001).

The median concentration of Pb in urine was higher in the patients with ALS than in the HCs. The patients with ALS had a median concentration of 0.58 µg/L (0.13–1.09 [percentile 2–percentile 75, P25–P75]) while the controls’ median concentration was 0.05 µg/L (0.05–0.05 [P25–P75]). The differences were statistically significant (*p* < 0.001) (Figure 1, Table 2).

### 3.2. Manganese (Mn)

For Mn, in most participants, the levels were not detected in urine, and therefore the median was the same in both patients and controls, equivalent to the minimum level (the value assigned when it was not detectable). However, there were differences in the average concentrations, being 0.43 µg/L in the patients with ALS and 0.25 µg/L in the HCs. The differences were statistically significant (*p* = 0.043) (Figure 1, Table 2).

### 3.3. Selenium (Se)

The average concentration of Se in urine was higher in the patients with ALS than in the HCs. The patients with ALS had an average concentration of 35.14 µg/L (SD 12.68) while the controls’ average concentration was 27.73 µg/L (SD 16.39). The differences were not statistically significant (*p* = 0.146) (Figure 1, Table 2).

### 3.4. Copper (Cu)

For Cu, the median concentration was also higher in the patients with ALS than in the HCs. The patients with ALS had a median concentration of 8.62 µg/L (6.28–16.09 [P25–P75]) while the controls’ median concentration was 5.06 µg/L (2.94–7.37 [P25–P75]). The differences were statistically significant (*p* = 0.007) (Figure 1, Table 2).

### 3.5. Zinc (Zn)

The latest metal studied was Zn. The patients with ALS had a median concentration of 346.36 µg/L (201.53–607.53 [P25–P75]) while the HCs’ median concentration was 253.59 µg/L (166.89–428.46 [P25–P75]). The differences were not statistically significant for this metal (*p* = 0.835) (Figure 1, Table 2).

## 4. Discussion

Sporadic ALS, which accounts for over 90% of cases, is widely regarded as the outcome of complex interactions among genetic factors, aging, and environmental exposures. However, the specific contribution of each component has not been elucidated yet [4]. Given the rapid increase in the disease incidence, there is a pressing need to better characterize the disease pathogenesis, including the role of environmental factors. Within this category, exposure to toxicants, and particularly to heavy metals, has been widely assessed, with discordant results. In this study, urine concentrations of five different heavy metals were examined in search of a possible association between these chemical substances and sALS. Regarding the study of heavy metals and motor neuron degeneration, some authors have assessed metal exposures based on diet type and food composition [23]. Other investigators have focused on the presence of metals in rivers to quantify the exposure of nearby populations [24]. Additionally, some studies have assessed the relationship between ALS distribution and air pollutants [25,26], specifically measuring the presence of heavy metals in the air [27]. Along these lines, our group reported a positive correlation between ALS mortality and air lead levels in Spain [28]. Concerning studies on metal exposure, the complementary approach of analyzing biological samples provides a more accurate assessment of individual exposures. In this context, Violi et al. used cerebrospinal fluid samples from patients with ALS and measured the levels of trace metals—Cu, iron (Fe), and Mn—describing a possible positive association with Cu [29]. Royce-Nagel et al. analyzed hair samples from patients and evaluated the levels of several metals, including vanadium, aluminum (Al), magnesium (Mg), and Mn [30]. Only Al concentration was found to be higher in patients compared to HCs, but the differences were not statistically significant. The authors concluded that environmental metal exposure could not be excluded as a factor in ALS development and progression, since hair samples only reflect exposures during their growth period and may miss earlier exposures [30].

Urine analysis reflects only recent exposures. Therefore, if metal sources are linked to stable factors, such as residence, diet, or long-term activities, the results can be considered representative. In contrast, more distant exposures are unlikely to be captured, since past habits may not be reflected in urine samples. In the literature, studies using urine samples in patients with ALS are very limited. In fact, Farace et al., in a meta-analysis of Pb bioaccumulation in patients with ALS, could not include urine data due to insufficient literature [31]. Bocca et al. analyzed levels of Al, Cd, Hg, Mn, and Pb and found no statistically significant differences between patients with ALS and HCs [32]. In contrast, Jang et al., in a case-control study, concluded that metals (including Cu, Se, and Zn) in plasma and urine were associated with increased ALS risk and reduced survival. These findings seemed to be independent of genetic risk and correlated with occupational and non-occupational metal exposures [33]. Although these authors did not include all the metals analyzed in our study, their findings regarding Cu are consistent with those observed in our analysis.

In our study, patients with ALS showed significantly higher urinary concentrations of Pb and Cu compared to control subjects. Exposure to these compounds may result from various sources, including diet and drinking water, supplement intake, or environmental pollution, among others [34]. Importantly, Pb and Cu are two heavy metals most related to ALS by far [35]. The mechanisms by which these metals could induce motor neuron degeneration have been documented in in vitro experiments and in preclinical models of the disease [36]. Exposure to these metals increases oxidative stress levels, disrupts mitochondrial function, and interferes with protein folding—all of which are key pathogenic events in ALS [37,38].

Increased urinary concentrations of Pb and Cu in patients with ALS may result from multiple factors, including higher metal external exposure, differences in clearance capacity, and/or metabolic alterations. As both patients with ALS and controls belonged to the same healthcare area, substantial differences in exposure to primary sources, such as public water supply or industrial activity, would not be expected. Nevertheless, it cannot be excluded that exposures related to individual lifestyle habits may have contributed, at least in part, to the observed findings. There is no evidence that patients with ALS exhibit impaired clearance of heavy metals compared with the general population [35]. In this context, studies reporting elevated metal levels suggest that the differences observed in patients are more likely due to external exposures and dysregulation of metal metabolism rather than to defects in metal elimination [35]. Regarding Pb metabolism, Kamel et al. investigated whether polymorphisms in the aminolevulinic acid dehydratase (ALAD) gene, which may modulate susceptibility to Pb exposure, were associated with ALS risk. Notably, certain ALAD variants were linked to lower blood Pb levels, suggesting that genetic susceptibility via ALAD polymorphisms may influence ALS risk, possibly through mechanisms related to internal lead exposure [39]. Overall, these findings support a role for Pb and Cu in disease pathogenesis and highlight potential novel therapeutic targets.

Regarding Mn, although differences were observed, concentrations were below the detection limit in most participants, limiting the validity of these results. Potential mechanisms by which elevated Mn could contribute to motor neuron degeneration include astroglial toxicity, mitochondrial dysfunction, and increased oxidative stress [40].

Finally, despite prior evidence in the literature, no significant differences were observed in urinary Se and Zn concentrations between patients with ALS and HCs. Of these, the association with Se is the most consistently supported [4]. Notably, most reported links between ALS and Se arise from external contamination via exposure to seleniferous water sources [41], a scenario not present in the geographical area of the present study.

This pilot study included a limited number of patients, and its findings require validation in external cohorts. Both bulbar and spinal forms of ALS were represented, although no differences in heavy metal concentrations were observed between these subgroups. In addition, although participants with a prior history of supplement use or potentially chelating treatments were excluded, dietary habits and occupational exposures were not systematically evaluated. This omission cannot be excluded as a potential source of bias and may have influenced the study results. Moreover, a potential limitation of our study is that absolute concentrations of heavy metals were reported without adjustment for urinary dilution. While creatinine correction was not applied, due to the well-recognized reduction of creatinine levels in patients with ALS [42], the absence of adjustment—whether by creatinine or specific gravity—reduces comparability with previous research [43,44]. In addition, variability in urine dilution across individuals may have introduced bias, either by exaggerating apparent case-control differences or by obscuring true associations [45,46]. This methodological constraint should be considered when interpreting our findings and highlights the importance of standardized approaches for urinary biomarker normalization in future studies. In this regard, the absence of dilution adjustment might have contributed to the Mn finding, affected the magnitude of differences in Pb and Cu, and obscured potential associations with Se and Zn. As a final limitation, as ALS progresses, patients typically experience increasing disability, which may limit physical activity and alter dietary habits and hydration status [47]. Importantly, the study also has several notable strengths: all sample collection, processing, and analysis were conducted at a single time point; the study population belonged to the same healthcare area, likely sharing a similar genetic background; and the controls were individually matched to each case.

Overall, this pilot study identified elevated urinary concentrations of Pb, Mn, and Cu in patients with ALS compared with non-genetically related HC from the same geographic area but residing in separate households. These findings may reflect higher environmental exposure to these metals, although altered metabolic or excretory pathways in patients with ALS could also contribute. While causality cannot be inferred, the results highlight a potential pathogenic role of heavy metals in ALS. Large-scale studies are needed to further clarify the specific contribution of these toxicants to ALS pathogenesis.

## Figures and Tables

**Table 1:** Main clinical-demographic data of patients and healthy controls.

	Sex	Age	Type of ALS	ALSFRS-R at Sampling	Environment
Patients with ALS					
Patient 1	Female	51	Spinal	11	Urban
Patient 2	Male	62	Spinal	45	Urban
Patient 3	Male	84	Spinal	13	Rural
Patient 4	Female	75	Bulbar	26	Urban
Patient 5	Male	71	Spinal	45	Urban
Patient 6	Male	67	Spinal	33	Rural
Patient 7	Male	81	Spinal	44	Rural
Patient 8	Male	62	Spinal	41	Urban
Patient 9	Female	68	Spinal	43	Rural
Patient 10	Female	69	Spinal	37	Urban
Patient 11	Female	69	Bulbar	30	Urban
Patient 12	Female	82	Spinal	39	Rural
Patient 13	Male	69	Spinal	33	Rural
Patient 14	Female	65	Spinal	17	Urban
Healthy controls (HC)					
HC 1	Female	64	-	-	Urban
HC 2	Male	67	-	-	Urban
HC 3	Female	61	-	-	Urban
HC 4	Female	69	-	-	Urban
HC 5	Male	70	-	-	Urban
HC 6	Male	72	-	-	Urban
HC 7	Female	71	-	-	Urban
HC 8	Male	64	-	-	Rural
HC 9	Female	84	-	-	Rural
HC 10	Female	69	-	-	Rural
HC 11	Female	77	-	-	Rural
HC 12	Female	62	-	-	Rural
HC 13	Male	63	-	-	Rural
HC 14	Male	69	-	-	Rural
HC 15	Female	62	-	-	Rural
HC 16	Male	68	-	-	Rural
HC 17	Male	80	-	-	Rural
HC 18	Female	74	-	-	Urban
HC 19	Female	75	-	-	Urban
HC 20	Male	66	-	-	Urban
HC 21	Male	65	-	-	Urban
HC 22	Female	68	-	-	Urban
HC 23	Female	62	-	-	Urban
HC 24	Male	63	-	-	Urban
HC 25	Male	67	-	-	Urban
HC 26	Male	92	-	-	Urban
HC 27	Female	84	-	-	Rural
HC 28	Male	66	-	-	Rural

ALSFRS-R: Revised Amyotrophic Lateral Sclerosis Functional Rating Scale.

**Table 2:** Comparative description of the values of the five metals studied detected in urine in patients with ALS (cases) and healthy controls.

	Pb		Mn		Se		Cu		Zn	
	ALS	HC	ALS	HC	ALS	HC	ALS	HC	ALS	HC
Average (μg/L)	0.69	0.13	0.43	0.25	35.14	27.73	10.36	6.18	467.28	302.73
Median (μg/L)	0.58	0.05	0.25	0.25	34.25	25.05	8.62	5.06	346.36	253.59
P25 (percentile 25)	0.13	0.05	0.25	0.25	22.88	15.55	6.28	2.94	201.53	166.89
P75 (percentile 25)	1.09	0.05	0.25	0.25	45.6	34.45	16.09	7.37	607.53	428.46
Number of samples below detection limit	2	25	12	28	0	0	0	0	0	0
Test Statistic	49.00		168.00		1.48		94.50		162	
*p*-value	<0.001		0.043		0.146		0.007		0.835	

ALS: Amyotrophic Lateral Sclerosis; HC: Healthy controls. The test statistic is U for all the metals except for Selenium, for which it is t.

## Data Availability

The data presented in this study are partially available due to ethical restrictions. Some of the data contain sensitive patient information for which no consent was obtained for sharing; therefore, only de-identified and non-restricted data can be made available upon reasonable request from the corresponding author.

## Abbreviations

The following abbreviations are used in this manuscript:

ALS	Amyotrophic lateral sclerosis
ALSFRS-R	Revised ALS functional rating scale
Al	Aluminium
Cu	Copper
fALS	Familial Amyotrophic Lateral Sclerosis
HCs	Healthy controls
ICP-MS	Inductively Coupled Plasma Mass Spectrometry
Pb	Lead
Mg	Magnesium
Mn	Manganese
P25–P75	Percentile 25–Percentile 75
sALS	Sporadic Amyotrophic Lateral Sclerosis
SD	Standard deviation
Se	Selenium
Zn	Zinc
